# Isotype Heterojunction-Boosted CO_2_ Photoreduction to CO

**DOI:** 10.1007/s40820-022-00821-9

**Published:** 2022-03-12

**Authors:** Chaogang Ban, Youyu Duan, Yang Wang, Jiangping Ma, Kaiwen Wang, Jiazhi Meng, Xue Liu, Cong Wang, Xiaodong Han, Guozhong Cao, Liyong Gan, Xiaoyuan Zhou

**Affiliations:** 1 College of Physics and Center of Quantum Materials and Devices, Chongqing University, Chongqing, 401331 People’s Republic of China; 2 State Key Laboratory of Coal Mine Disaster Dynamics and Control, Chongqing University, Chongqing, 401331 People’s Republic of China; 3 Beijing Key Laboratory of Microstructure and Property of Advanced Materials, Beijing University of Technology, Beijing, 100024 People’s Republic of China; 4 Analytical and Testing Center, Chongqing University, Chongqing, 401331 People’s Republic of China; 5 Department of Materials Science and Engineering, University of Washington, Seattle, WA 98195 USA

**Keywords:** Isotype heterojunction, g-C_3_N_4_, CO_2_ photoreduction, Charge dynamics, Reaction mechanism

## Abstract

Photocatalytic conversion of CO_2_ to high-value products plays a crucial role in the global pursuit of carbon–neutral economy. Junction photocatalysts, such as the isotype heterojunctions, offer an ideal paradigm to navigate the photocatalytic CO_2_ reduction reaction (CRR). Herein, we elucidate the behaviors of isotype heterojunctions toward photocatalytic CRR over a representative photocatalyst, g-C_3_N_4_. Impressively, the isotype heterojunctions possess a significantly higher efficiency for the spatial separation and transfer of photogenerated carriers than the single components. Along with the intrinsically outstanding stability, the isotype heterojunctions exhibit an exceptional and stable activity toward the CO_2_ photoreduction to CO. More importantly, by combining quantitative in situ technique with the first-principles modeling, we elucidate that the enhanced photoinduced charge dynamics promotes the production of key intermediates and thus the whole reaction kinetics.

**Supplementary Information:**

The online version contains supplementary material available at 10.1007/s40820-022-00821-9.

## Highlights

- The g-C_3_N_4_ isotype heterojunction was synthesized for photocatalytic CO_2_ reduction, which exhibits an impressive activity and outstanding stability.
- The isotype heterojunction presents more favorable charge separation and transfer performance than the single components.
- The enhanced photogenerated charge dynamics in isotype heterojunction facilitates the production of key intermediates and thus the whole reaction kinetics.

**Supplementary Information:**

The online version contains supplementary material available at 10.1007/s40820-022-00821-9.

## Graphical abstract

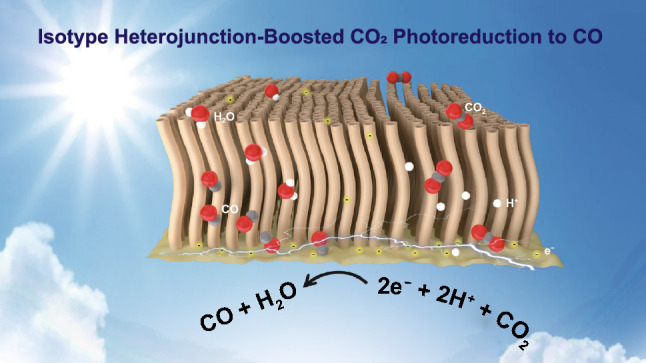

## Introduction

The conversion of CO_2_ into value-added fuels and feedstock is increasingly attractive due to the growing urgency to advance a sustainable carbon–neutral economy [1]. Light-driven CRR over photocatalysts is one of the most promising conversion technologies since it can proceed under relatively mild conditions, which facilitates its resource utilization [2–10]. In the past decades, great progress has been made. The proper design of junction photocatalysts enables superior conversion efficiency [11–14]. Moreover, several fundamental principles have been identified to rationalize the superiority. These primarily include the narrowed band gaps and particularly the feasibility and effectiveness for the spatial separation and transfer of photogenerated electron–hole pairs [12, 14]. The former allows wide light-absorption range [12, 15], and the latter is ascribed as the synergy between the internal electric field and the band alignment at the interface [16–19]. As a result of the favorable attributes, enormous efforts are continuously undertaken in the design and investigation of junction photocatalysts for CO_2_ photoreduction [11–14, 16–25].

This avenue is potentially extended by constructing isotype heterojunctions between two different phases with staggered gaps of an identical substance [18–25]. Particularly, due to the similar crystal lattices and electronic structures, isotype heterojunctions exhibit a superior stability [20], close contact, native compatibility and thus lower potential barrier for charge migration than the heterotype counterparts [21, 22]. It has been reported that the isotype heterojunctions of rutile-/anatase-TiO_2_ and monoclinic/tetragonal BiVO_4_ exhibit superior photocatalytic properties [23–25]. More recently, a high solar-to-hydrogen conversion efficiency of 1.16% was reported over an isotype heterojunctions consisting of boron-doped and nitrogen-deficient g-C_3_N_4_ [19]. These encouraging achievements indicate the superior photocatalytic properties of isotype heterojunctions, which are highly desirable for improving the performance of photocatalytic CRR [26–30]. However, up to date, no studies have reported photocatalytic CRR over isotype heterojunctions. Uncovering the detailed reaction mechanism can provide new insights into modulating the catalytic behavior by junction engineering in photocatalytic CRR materials [31–35]. Particularly, it is well known that enhanced photogenerated carrier dynamics enables higher performances. However, a long-standing question in photocatalysis is that how such enhancement actually contributes to the overall reaction kinetics [31, 33–35].

In this work, aiming at a thorough picture of how the enhanced photoinduced carrier dynamics affects photocatalytic CRR, we comparatively study the photocatalytic properties of the isotype heterojunctions and the single components. The isotype heterojunctions were designed and synthesized based on a prototypical photocatalyst, g-C_3_N_4_ [36–38]. Detailed synthesis processes of the samples can be found in Supporting Information. Impressively, g-C_3_N_4_ with isotype heterojunctions exhibits largely boosted photocatalytic CRR performance. Furthermore, it is revealed that the formation of isotype heterojunctions enhances the separation and transfer of photogenerated charges. Particularly, such enhancement would directly accelerate the production of key intermediates and thus the whole reaction kinetics.

## Experimental Section

### Synthesis of Samples

The analytical-grade chemical reagents of thiourea (CH_4_N_2_S) and melamine (C_3_H_6_N_6_) were obtained from Shanghai Aladdin Bio-Chem Technology Co., LTD. Ethanol was purchased from Chengdu Kelong Chemical Co., Ltd. The preparation of supramolecular is as follows: firstly, 2 g melamine was mixed with 40 mL deionized water. Subsequently, it was transferred to a 50-mL Teflon-lined autoclave and placed in a 180 °C oven for 10 h. The hydrothermal products were washed several times and dried overnight at 60 ℃. Synthesis of g-C_3_N_4_ isotype heterojunction (ICN). Typically, 0.7 g of supramolecular precursor was mixed with different amounts of thiourea (0.1, 0.4, 0.7, 1.0 and 1.3 g) in 15 mL deionized water and stirred overnight at 60 °C. The obtained molecular composite precursors were heated to 550 °C and maintained for 4 h. Finally, based on the amount of thiourea, the resultant products were denoted as ICN-1, ICN-2, ICN-3, ICN-4 and ICN-5. The single components g-C_3_N_4_ were synthesized by calcining supramolecular and thiourea using the same calcination process as ICN, respectively, marked as MCN and TCN.

### Material Characterization

X-ray diffraction (XRD) data were recorded by a PANalytical X’pert diffractometer to determine the crystal structures of the fabricated samples. Fourier transform infrared (FTIR, Nicolet iS50) spectra were used to determine the functional groups of photocatalysts. The optical properties of the photocatalysts were surveyed by UV–Vis spectroscopy (Shimadzu UV-3600). X-ray photoelectron spectroscopy (XPS, Thermo Fisher Scientific ESCALAB250Xi) was employed to investigate the surface structure and composition of photocatalysts. Transmission electron microscopy (TEM, Thermo Fisher Scientific Talos F200S) and scanning electron microscopy (SEM, Thermo Fisher Scientific Quattro S) were carried out to explore the microstructure and morphology of photocatalysts. Nitrogen adsorption/desorption isotherms were measured on Quadrasorb 2MP full-automatic specific surface aperture analyzer; the specific surface area and pore size distribution curves were gained by the Brunner–Emmet–Teller (BET) method. Fluorescence spectrometer (FLS1000) was carried out to record the time-resolved fluorescence emission spectra (TRPL) at excitation wavelength of 395 nm. Photoluminescence spectra (PL) were obtained by a steady-state fluorescence spectrometer (Shimadzu RF-6000) at room temperature. The intermediate species in photocatalytic CO_2_ reduction were studied by in situ diffuse reflectance infrared Fourier transform spectroscopy (in situ DRIFTS) (Bruker Vertex 70 V).

### Photocatalytic CO_2_ Reduction

The light source was a 300-W Xe lamp with an AM 1.5 filter (Beijing Perfectlight Technology Co., Ltd. PLS-SXE300/300DUV Xenon lamp source. The light intensity on the catalyst surface is 103.2 mW cm^−2^). The source of CO_2_ for the photocatalytic reduction reaction is highly pure CO_2_ (99.999%). To specify, 5 mg photocatalysts were ultrasonically dispersed in 2 mL deionized water at a quartz culture dish and dried at 60 °C. Then, the quartz culture dish containing photocatalysts was placed in a 150-mL reaction chamber (Beijing Perfectlight Technology Co., Ltd. PLR MFPR-I Multifunctional photochemical reactor) and 200 μL deionized water was dripped around the dish. The CO_2_ pressure and temperature were maintained at 105 kPa and ambient temperature 25 °C. Finally, the reaction chamber was illuminated. The generated gas products were determined by a gas chromatograph (GC-2014C, SHIMADZU).

### Photoelectrochemical Measurements

The electrochemical impedance spectroscopy (EIS), photocurrent, and Mott–Schottky plots were recorded on the electrochemical workstation (Zahner CIMPS-2) with a standard three-electrode cell. The working, counter and reference electrodes were glassy carbon electrode deposited with photocatalysts, Pt plate and Ag/AgCl, respectively. The 0.5 M Na_2_SO_4_ aqueous solution was served as an electrolyte. The working electrode was prepared as follows: 5 mg photocatalysts were ultrasonically dispersed in 50 μL Nafion and 950 μL ethanol for 10 min, which resulted in a uniform dispersion of the suspension. Then, 10 μL suspension was dropped onto the glassy carbon electrode and dried naturally.

## Results and Discussion

### Chemical Structure and Morphology

**Fig. 1:**
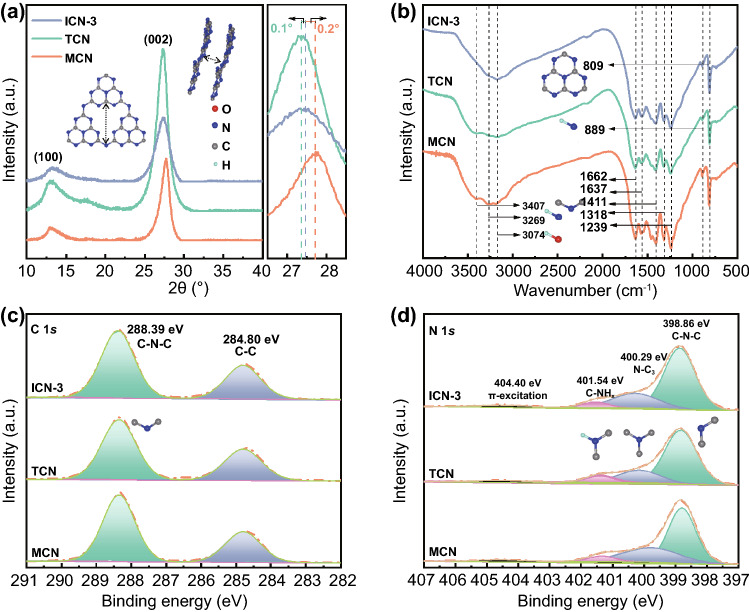
**a** XRD patterns, **b** FTIR spectra, **c** XPS for C 1s and **d** N 1s spectra of ICN-3, TCN and MCN

The g-C_3_N_4_ with isotype heterojunctions (ICN) was fabricated by calcining the mixture of supramolecular precursor and thiourea. Supramolecular precursors were derived from melamine by hydrothermal reaction. The supramolecular was formed through the self-assembly of melamine molecule and cyanuric acid molecule via hydrogen bonding. A series of samples were synthesized with various contents of thiourea while keeping the amount of supramolecular precursors constant, named ICN-*x* (*x* = 1, 2, 3, 4, 5) (Fig. S1). In comparison, the single components g-C_3_N_4_ were, respectively, synthesized by calcining thiourea and supramolecular precursors, which were marked as TCN and MCN. XRD technique was applied to determine the phase structures of ICN-*x* (*x* = 1, 2, 3, 4, 5), MCN and TCN. As shown in Figs. 1a and S2a, the XRD patterns of all samples display two characteristic diffraction peaks at around 13.1° and 27.3°, corresponding to the in-plane structural repeating motif of the tri-triazine units [39] and the interlayer reflection of a graphite-like structure [40]. Clearly, there is a remarkable difference (0.3°) between the two (002) peaks of TCN and MCN, suggesting their different crystal plane spacing. This discrepancy would lead to staggered band strucures between TCN and MCN, which potentially form isotype heterojunctions [16]. The functional groups of ICN-*x* (*x* = 1, 2, 3, 4, 5), MCN and TCN were further analyzed by FTIR spectra (Figs. 1b and S2b), in which four groups of characteristic bonds are obviously observed. They can be sequentially assigned to the breathing mode of the heptazine heterocyclic ring units (809 cm^−1^), the deformation of N–H bonds (889 cm^−1^) [40, 41], the stretching vibration modes of N−C=N (from 1239 to 1662 cm^−1^), the vibration of N–H bonds in the amine groups (broad peak at 3269 and 3407 cm^−1^) and the adsorbed H_2_O molecules (3074 cm^−1^) [39, 42]. These structural features are consistent well with previous studies [21, 39], signifying the synthesis of the typical graphitic-like g-C_3_N_4_.

The chemical composition and element states were examined using XPS [39]. As shown in Fig. S2c, the overall signals are completely the same in ICN-3, MCN and TCN, and the C, N, and O elements can be identified. The sulfur element is hardly detected in TCN and ICN-3, suggesting its complete release during the heat treatment. Only one small peak appears at 532.1 eV for the O 1s spectra (Fig. S2d), which can be obviously attributed to the adsorbed H_2_O [43]. The C 1s signal (Fig. 1c) is fitted into two sets of peaks at 284.8 and 288.4 eV, corresponding to the C–C bond and C–N–C bond [42], respectively. The N 1s spectra (Fig. 1d) exhibit four peaks at 398.9, 400.3, 401.5, and 404.4 eV, which can be assigned to C–N–C, N–C_3_, C–NH_*x*_, and π excitations, respectively [44]. The XPS results clearly indicate that there exist C and N elements in the as-synthesized samples. In addition, energy-dispersive X-ray spectroscopy (EDX) elemental mapping of ICN-3 (Fig. S3c–e) reveals uniform distribution of C, N elements, being consistent with the XPS results.

**Fig. 2:**
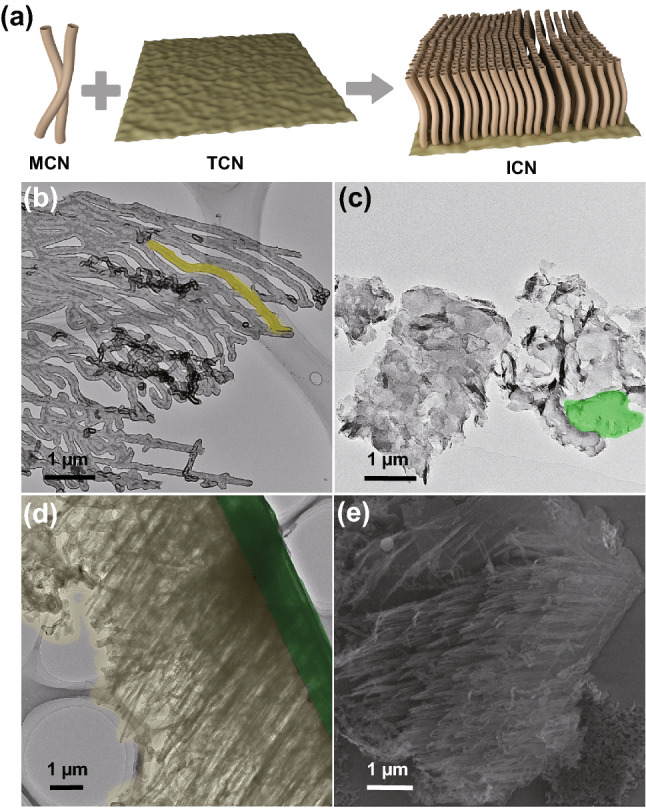
**a** Schematic diagram of ICN. TEM images of **b** MCN, **c** TCN and **d** ICN-3. **e** SEM images of ICN-3

The TEM images (Fig. 2b–d) of the three samples are compared. It is found that a nanotube-like structure occurs in MCN (Fig. 2b). The wall thickness, diameter and length are, respectively, ~ 20 nm, ~ 50 nm and ~ 2.5 μm. The diameter is further estimated to be 52.5 nm by the average pore diameter analysis (Fig. S3a). In comparison, the TCN sample obviously displays nanosheet-like structure (Fig. 2c). Its lateral size is up to several micrometers, and the average pore diameter is 29.3 nm. Upon in situ growing the nanotubes on the nanosheets, the TEM image (Figs. 2d and S3c–g) reflects that the two morphological features are largely inherited in the resultant ICN-3 sample. Clearly, the HRTEM analysis indicates that the nanotube arrays closely grow on the surfaces of nanosheets (Figs. S3g and 2a). Furthermore, SEM characterization (Fig. 2e) also displays the tight packing between the two phases. These results strongly indicate the successful synthesis of g-C_3_N_4_ isotype heterojunctions. In line with these observations, as demonstrated in the N_2_ adsorption/desorption isotherms curves, the specific surface areas of ICN-3, MCN and TCN are calculated to be 22.23, 23.00 and 9.38 m^2^ g^−1^, respectively (Fig. S3b and Table S1), suggesting that the hybrid sample is identical to its single-component systems in terms of the specific surface area and the number of active centers.

### Optical Properties and Band Structures

**Fig. 3:**
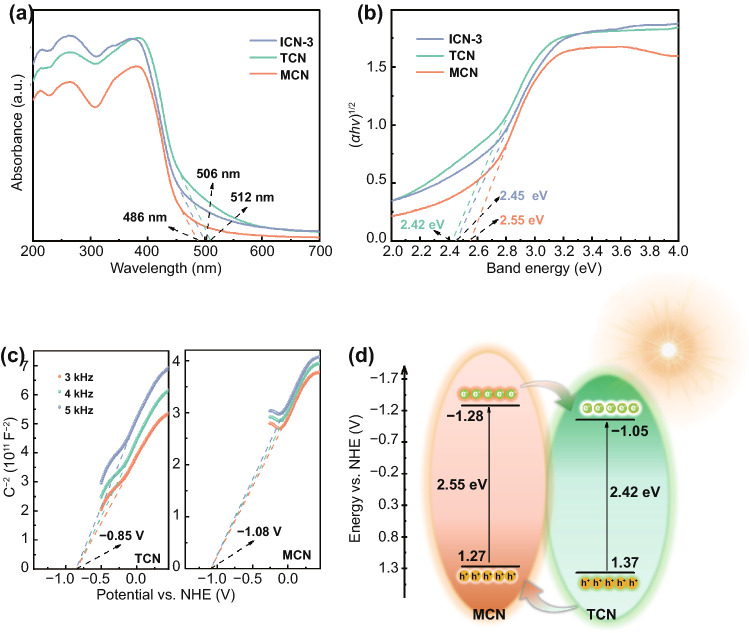
**a** UV-DRS spectra. **b** Corresponding Tauc plots of as-obtained photocatalysts. **c** The Mott–Schottky plots of TCN and MCN. **d** Schematic of electrons–holes separation and transfer at the interface between TCN and MCN

The optical properties and band structures of the synthesized samples were determined by UV-visible diffuse reflectance spectrum (DRS). As displayed in Fig. 3a, MCN and TCN exhibit an absorption edge of ~ 486 and ~ 512 nm, corresponding to an intrinsic band gap of 2.55 and 2.42 eV, respectively. The difference between the two systems provides great potential to design isotype heterojunctions with a well-matched band structure [16, 45]. Moreover, from the intercept of the tangents to the Mott–Schottky plots (Figs. 3c and S4b), the respective conduction bands of the two individual components were estimated to be − 1.28 and − 1.05 V versus the normal hydrogen electrode (NHE) at pH = 7. Accordingly, corresponding valence bands were calculated to be 1.27 and 1.37 V versus NHE for MCN and TCN, respectively. Meanwhile, the valence band potentials of TCN and MCN are 2.17 and 2.04 V according to XPS-VB spectra (Fig. S5). Based on these values, the band positions are elucidated and displayed in Fig. 3d. Notably, a staggered band alignment is formed, giving rise to a conduction band offset of 0.23 eV and a valence band offset of 0.10 eV.

As shown in Fig. 3a, b, the band absorption edges of MCN, ICN-3, and TCN originate from the π–π* electron transition upon irradiation [44, 46]. ICN-3 exhibits approximately an averaged light-harvesting ability of MCN and TCN and a slight red shift in comparison with MCN (506 vs. 486 nm). TCN has the smallest bandgap and thus the widest light-absorption range. Upon the formation of isotype heterojunctions, ICN-3 inherits the band features partially from TCN. Accordingly, the incorporation of TCN should be responsible for the obtained slight red shift in comparison with MCN. Moreover, the intrinsic band gap of ICN-3 was determined to be 2.45 eV and the conduction band of ICN-3 (Fig. S4b) was estimated to be ~ − 1.25 V. The two values both lie between those of the two single phases, indicating the intimate electronic coupling at the interfaces and thus confirming the formation of heterojunction with a well-matched band structure [16]. As a result of these features, it is expected that photogenerated charges can be effectively separated and transferred at the interfaces of ICN-3, guaranteeing more photogenerated charges for improved photocatalytic activity.

### Photogenerated Charge Dynamics Mechanism

**Fig. 4:**
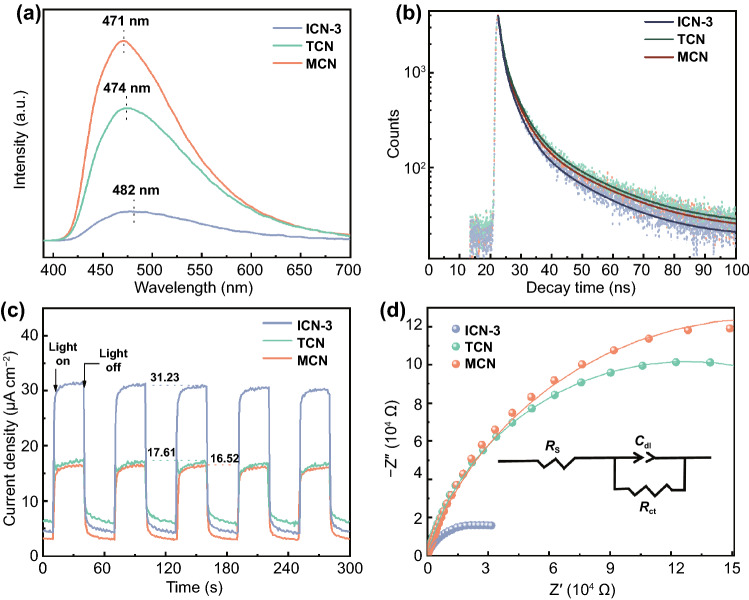
**a **Steady-state photoluminescence spectroscopy, **b** time-resolved photoluminescence spectroscopy, **c** transient photocurrent response, and **d** electrochemical impedance spectroscopy of photocatalysts

The photogenerated charge dynamic processes of photocatalytic reactions can be divided into two main steps: the excitation and the transfer or recombination of electrons and holes [11–13, 17]. Particularly, the latter is of crucial importance as it determines whether the photo-excited charges can be effectively utilized for subsequent reactions. Thus, to obtain a deep understanding of carrier dynamics behaviors in the presence of isotype heterojunctions, a series of advanced techniques were applied including the steady-state photoluminescence (PL) spectroscopy, TRPL spectroscopy, transient photocurrent response, and EIS. As clearly seen in Fig. 4a, ICN-3 exhibits a significantly lower band-to-band emission peak at 482 nm than those of the single counterparts. Its PL quenching intensity is estimated to be as low as 15% and 22% of that of MCN and TCN, respectively. To gain more insight into the photogenerated charges dynamics, the luminescence decay curves are fitted exponentially with a third order, and the results are shown in Fig. 4b, from which the photo-excited charge short lifetimes of *τ*_1_ and *τ*_2_ as well as the long lifetime of *τ*_3_ are, respectively, obtained and used to further calculate the average lifetime *τ*_ave_ **(**Table 1) [47]. Notably, ICN-3 displays a shorter *τ*_ave_ of 7.79 ns than both MCN (8.95 ns) and TCN (9.06 ns). The much shorter fluorescence lifetime implies the existence of effective electron transfer channels from MCN to TCN in ICN-3, indicating that the isotype heterojunctions efficiently speeds up the carrier transfer and utilization [39]. Consequently, the charge transfer rate (*k*_CT_) and efficiency (*η*_CT_) at the interface for ICN-3 were calculated (Eqs. S1 and S2) to be 1.80 × 10^7^ s^−1^ and 13% [48, 49], respectively, demonstrating the effective charge transfer from MCN to TCN. These results clearly indicate that the formed isotype heterojunctions greatly facilitate the spatial separation and transfer of photogenerated charges and thus reduce the recombination rate of the carries.

**Table 1:** The photogenerated charge lifetime (*τ*_1_, *τ*_2_, *τ*_3_ and *τ*_ave_), transfer rate (*k*_CT_), transfer efficiency (*η*_*C*_), photogenerated charge transfer resistance (*R*_CT_) and photocurrent current density (*I*_*P*_) of samples

Samples	*τ*_1_ (ns)	*τ*_2_ (ns)	*τ*_3_ (ns)	*τ*_ave_ (ns)	*k*_CT_ (s^−1^)	*η*_CT_	*R*_CT_ (Ω)	*I*_*P*_ (μA cm^−2^)
MCN	1.13	4.16	19.75	8.91	–	–	3.33 × 10^5^	17.61
TCN	1.09	4.08	18.74	9.06	–	–	2.56 × 10^5^	16.52
ICN-3	1.03	3.76	17.25	7.79	1.80 × 10^7^	13%	3.94 × 10^4^	31.23

Transient photocurrent responses were comparatively measured to investigate how the presence of isotype heterojunctions influences the transfer ability of photogenerated charges; the results are shown in Fig. 4c. Clearly, the photocurrent current density (*I*_*p*_) of ICN-3 (31.23 μA cm^−2^) is nearly twice of that of MCN (17.61 μA cm^−2^) and TCN (16.52 μA cm^−2^), reflecting a considerably faster electron–hole separation and transfer kinetics. In addition, electrochemical impedance spectroscopy (Fig. 4d) reveals a significantly smaller semicircle radius than its single components. In order to quantify the impedance results, the Randle circuit model (Fig. 4d, inset) was established by fitting the EIS diagram. It is found that the photogenerated charge transfer resistance (*R*_ct_) in ICN-3 (3.94 × 10^4^ Ω) is only approximately ~ 10% of that in MCN (3.33 × 10^5^ Ω) and TCN (2.56 × 10^5^ Ω). These results provide further evidence for the remarkably enhanced electron–hole separation and transfer rate in ICN-3, being consistent well with PL and TRPL observations. Apparently, the promoted separation and transfer of photogenerated carriers should be attributed to the presence of isotype heterojunctions. As a result, the above-mentioned findings not only confirm the formation of intimate isotype heterojunctions in ICN-3, but also highlight the corresponding critical role in boosting the separation and transfer efficiency of photo-excited charges.

### Photocatalytic CO_2_ Reduction Activity

**Fig. 5:**
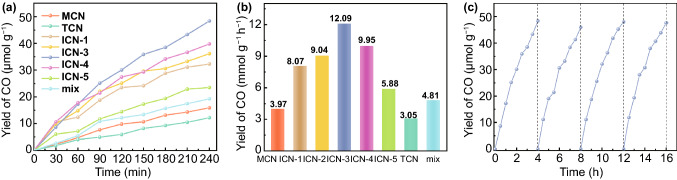
**a**, **b** CO production rates from photocatalytic CRR under irradiation. **c** Photocatalytic stability test of ICN-3 for 240 min in each cycle

An enhanced efficiency of charge separation and transfer implies a higher accessibility and utilization of the photogenerated carriers and thus potentially a superiority of photocatalytic performance. To this end, photocatalytic CRR was tested under the condition of ambient temperature and simulated solar irradiation, as shown in Fig. 5a. We have carried out the measurement of various gases (i.e., CO, CH_4_, C_2_H_6_, C_2_H_4_, C_2_H_2_, H_2_, and O_2_) and liquids (i.e., HCOOH, CH_3_OH, CH_3_COOH, and CH_3_CH_2_OH). It was found that CO is the only product for all the samples and the yield monotonically increases as a function of the irradiation time. On the other hand, as presented in Fig. 5b, MCN and TCN exhibit CO yield rates of 3.97 and 3.05 μmol g^−1^ h^−1^, respectively. It was found that the PL quenching intensity, photocurrent current density and the photogenerated charge transfer resistance of TCN are quite close to those of MCN, indicating that these factors contribute little to the performance difference between TCN and MCN. In contrast, the surface area and the conduction band potential of MCN are superior over those of TCN and thus should be responsible for the relative higher performance of MCN.

Clearly, the formation of isotype heterojunctions in ICN boosts the photocatalytic CO yields to a great extent. Moreover, as the amount of TCN increases, the photocatalytic performance remarkably improves and the CO yield rate reaches a maximum of 12.09 μmol g^−1^ h^−1^ in ICN-3, which is among the top value for g-C_3_N_4_-based CO_2_ reduction photocatalysts (Table S2). However, further increasing the concentration of TCN deteriorates the performance. According to the TEM (Figs. S6a, b and 2d), as the concentration of TCN increases from ICN-1 to ICN-3, the samples show that the nanotube arrays closely grow on the surfaces of nanosheets. However, upon further increasing the concentration of TCN, the morphology of ICN-4 and ICN-5(Fig. S6c, d) collapses, demonstrating that the presence of excess TCN degrades the concentration of isotype heterojunctions [45, 50]. In addition, we have also characterized CRR behavior of the mechanically mixed sample and a CO yield of 4.81 μmol g^−1^ h^−1^ was obtained. This is remarkably lower than that of ICN-3, collectively indicating the pivotal role of isotype heterojunctions in promoting CO_2_ photoreduction to CO.

Photocatalytic durability is another important criterion for evaluating the CO_2_ reduction catalysts. As shown in Fig. 5c, there is no apparent decline after 960 min during the cycling test, demonstrating a decent stability. Furthermore, no obvious changes were observed in the XRD, SEM and TEM (Fig. S7a–c) characterizations of ICN-3 after the duration of 960 min, indicating its good structural stability. In order to further determine the carbon source of the resultant products, carbon dioxide carrying the isotopes of ^13^C was used as the reactant and the product has been analyzed by gas chromatography–mass spectrometry (GC–MS). The results are shown in Fig. S8. It is found that the product is ^13^CO (m/z = 29), demonstrating that the photocatalytic product originates from CO_2_ reduction rather than the decomposition of the catalysts and other carbon-containing species.

### Photocatalytic CRR Reaction Mechanism

**Fig. 6:**
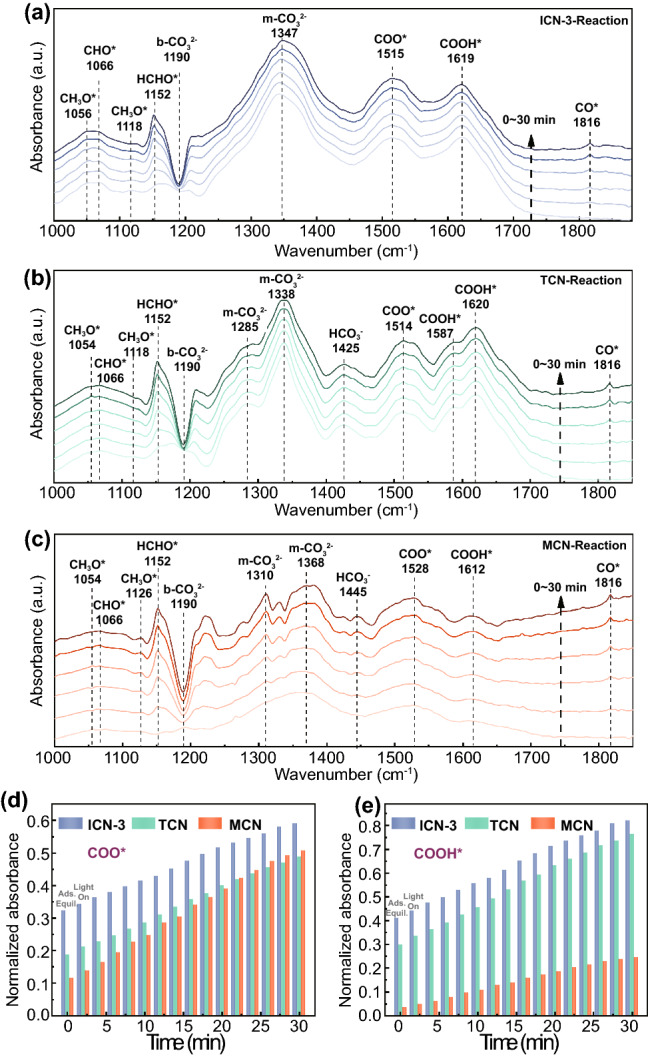
In situ DRIFTS analysis of photocatalytic CRR over **a** ICN-3, **b** TCN and **c** MCN. Normalized absorbance of **d** COO* and **e** COOH* on ICN-3, TCN and MCN

In order to understand the mechanism of photocatalytic CRR at a molecular level, the time-dependent evolutions of the reaction intermediates and products on ICN-3, TCN and MCN were monitored by in situ diffuse reflectance infrared Fourier transform spectroscopy (DRIFTS). As shown in Fig. 6a–c, the peaks at 1445 and 1425 cm^−1^ belong to HCO_3_^−^ [51, 52], while the peak at 1190 cm^−1^ is assigned to bidentate carbonates (b-CO_3_^2−^) [51]. In addition, a small amount of CH_3_O* (1056 and 1118 cm^−1^), CHO* (1066 cm^−1^), HCHO* (1152 cm^−1^) is detected [53, 54], resulting from the intermediates to the production of trace methane. Particularly, during the adsorption (Fig. S9a–c) and reaction period, the concentration of COO* (1515 cm^−1^) [55], m-CO_3_^2−^ (1310, 1347 cm^−1^) [51, 54], COOH* (1587 and 1619 cm^−1^) and CO* (1816 cm^−1^) is significantly increased [53, 55], indicating the dominance of these key intermediates. According to the in situ DRIFTS analysis, the possible reaction path for photocatalytic CRR is proposed as follows (the asterisks denote catalytically active sites).

1 $${\text{CO}}_{{2}} \left( {\text{g}} \right) \, + {\text{ H}}_{{2}} {\text{O}}\rightarrow{\text{2H}}^{ + } + {\text{ CO}}_{{3}}^{{{2} - }}$$

2 $$* \, + {\text{ CO}}_{{2}} \left( {\text{g}} \right)\rightarrow {\text{COO*}},{\text{ or }}* \, + {\text{2H}}^{ + } + {\text{ CO}}_{{3}}^{{{2} - }} \rightarrow {\text{H}}_{{2}} {\text{O }} + \, {\text{COO*}}$$

3 $${\text{COO*}} + {\text{ e}}^{ - } + {\text{ H}}^{ + } \rightarrow {\text{COOH*}}$$

4 $${\text{COOH*}} + {\text{ H}}^{ + } + {\text{ e}}^{ - } \rightarrow {\text{CO*}} + {\text{H}}_{{2}} {\text{O}}$$

5 $${\text{CO*}}\rightarrow {\text{CO}}\left( {\text{g}} \right) \,+ *$$

Furthermore, according to the local in situ DRIFTS in Fig. S10a, b, the normalized absorbance of the intermediates was calculated to investigate the impact of isotype heterojunctions on the reaction kinetics of CO_2_ reduction, selecting the well-known COO* and COOH* as representatives [56, 57]. As clearly shown in Fig. 6d, e, the absorbance of both species is remarkably stronger in the presence of isotype heterojunctions: the COO* and COOH* normalized absorbances on ICN-3 are, respectively, up to 1.21 and 3.34 times than that on the single components, indicating higher generation rates of the key intermediates. The superiority of photogenerated charge dynamics in ICN-3 should be responsible for the increased yield of both COO* and COOH* [57]. Moreover, as COOH* is formed by the protonation of COO*, the accelerated generation of COO* would also facilitate the production of COOH* [58]. These results clearly indicate that the presence of isotype heterojunctions enables faster reaction kinetics, leading to a significantly increased yield of CO.

**Fig. 7:**
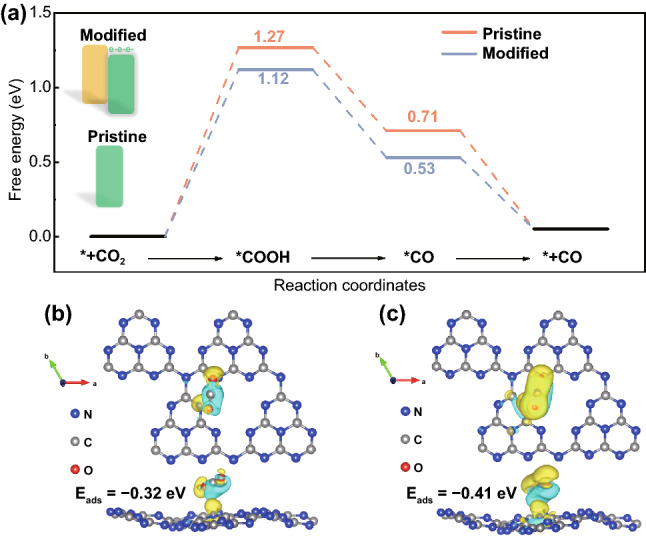
**a** Free energy profiles of photocatalytic CRR to CO. Difference charge density diagrams of CO_2_ adsorption on the **b** pristine and **c** modified g-C_3_N_4_. The isosurface value is 5 × 10^4^ e Å^−3^

To rationalize such remarkably enhanced catalyzing properties of the ICN-3 over the pristine g-C_3_N_4_ for the CO_2_ photoreduction and to discern the activity boost upon the formation of isotype heterojunctions, density functional theory calculations were performed to study the elementary reaction steps involved in photocatalytic CRR in the presence of isotype heterojunctions. The in situ DRIFTS observations show a significantly high yield of COOH* for TCN (Fig. 6e), reflecting that the reaction is likely to proceed overwhelmingly on the TCN side in ICN-3. Moreover, the band alignment (Fig. 3d) suggests that photogenerated electrons would transfer to the TCN side, rendering the nanosheet electron-rich. Accordingly, similar to previous studies [59], the effect of isotype heterojunctions was qualitatively investigated by a comparative study of CRR over a g-C_3_N_4_ monolayer with and without excess electrons. Specifically, the pristine g-C_3_N_4_ was adopted to model the TCN sample, and g-C_3_N_4_ modified by extra electrons was used to model the TCN side in the ICN-3 sample. Subsequently, CO_2_ adsorption on the two models was investigated and both O-end and C-end of CO_2_ were considered (Fig. S11a, b). It was found that the O-end configuration is unstable and would relax spontaneously to a C-end configuration. Clearly, the modified g-C_3_N_4_ exhibits much stronger CO_2_ adsorption, suggesting that the electron-rich feature of the TCN side in ICN-3 enhances the adsorption of CO_2_. Figure 7a presents the typical free energy diagram for the stepwise CO_2_ photoreduction to CO on the pristine and modified g-C_3_N_4_ [53]. It is apparent that the formation of COOH* is the most endothermic step on both modeled catalysts; this is similar to those on other g-C_3_N_4_-based photocatalysts [57, 60]. A comparison of the free energy profiles indicates that the path of CO_2_ photoreduction on the modified g-C_3_N_4_ is energetically smoother than that on the perfect one. Particularly, the formation of COOH* on the modified sample is remarkably less endothermic. This is also in accordance with the in situ DRIFTS observations. To gain a deep insight into the modification of excess electrons, the charge redistribution upon CO_2_ adsorption was visualized (Fig. 7b, c). Clearly, the modified system exhibits remarkably more charge transfer, interpreting the obtained stronger binding strength. These results clearly suggest that the resulting electron-rich feature due to the presence of isotype heterojunctions enables a higher intrinsic activity toward CO_2_ photoreduction.

## Conclusions

In summary, we have systematically investigated the photocatalytic CRR properties of g-C_3_N_4_ containing isotype heterojunctions. It has been demonstrated that the isotype heterojunctions enable a high separation and transfer efficiency of the photogenerated carriers in contrast to the single components. Combined with the intrinsic superior stability, the presence of isotype heterojunctions leads to a decent and stable activity toward the CO_2_ photoreduction to CO, which is among the top values for g-C_3_N_4_-based photocatalysts. Furthermore, it is revealed that the enhanced photogenerated charge dynamics could directly facilitate the yield of key intermediates and thus the whole reaction kinetics. This work provides vital insights into the design of high-performance CO_2_ reduction photocatalytic systems by engineering the microstructure of catalysts with heterostructures.

## Supplementary Information

Below is the link to the electronic supplementary material.

Supplementary file1 (PDF 1062 KB)
