# Supplementary material for: Isotype Heterojunction-Boosted CO2 Photoreduction to CO
Source: Nanomicro Lett. 2022 Mar 12;14:74. doi: 10.1007/s40820-022-00821-9 (PMC8918288; doi:10.1007/s40820-022-00821-9)
Supplement: Supplementary file 1 — Supplementary file1 (PDF 1062 KB) [file 40820_2022_821_MOESM1_ESM.pdf]

Supporting Information for

Isotype Heterojunction-Boosted CO_2 Photoreduction to CO

Chaogang Ban[1,†], Youyu Duan[1,†], Yang Wang[1], Jiangping Ma[1], Kaiwen Wang[3], Jiazhi Meng[1], Xue Liu[1], Cong Wang[3], Xiaodong Han[3], Guozhong Cao[5], Liyong Gan[1,2,*], Xiaoyuan Zhou[1,2,4,*]

[1]College of Physics and Center of Quantum Materials and Devices, Chongqing University, Chongqing 401331, P. R. China

[2]State Key Laboratory of Coal Mine Disaster Dynamics and Control, Chongqing University, Chongqing 401331, P. R. China

[3]Beijing Key Laboratory of Microstructure and Property of Advanced Materials, Beijing University of Technology, Beijing 100024, P. R. China

[4]Analytical and Testing Center, Chongqing University, Chongqing 401331, P. R. China

[5]Department of Materials Science and Engineering, University of Washington, Seattle, Washington 98195, USA

†Chaogang Ban and Youyu Duan contributed equally to this work

*Corresponding authors. E-mails: ganly@cqu.edu.cn (L.Y. Gan), xiaoyuan2013@cqu.edu.cn (X.Y. Zhou)

S1 Density Functional Theory Calculations

All calculations presented in this work were performed using the density functional theory within the Vienna Ab initio Simulation Package [S1, S2]. The projector augmented wave method was used to describe the ion-electron potential. The generalized gradient approximation with the Perdew-Burke-Ernzerhof functional was employed as the exchange correlation function [S3, S4]. The cutoff energy was set as 500 eV to ensure the accuracy and the DFT-D3 method was considered to correct the dispersion interactions [S5, S6]. To imitate CRR on the electron-rich g-C_3N_4, extra electrons were added in the system [S7]. The Brillouin zone was integrated using a Γ-centered 3×3×1 and 5×5×1 mesh respectively for geometry relaxation and electronic calculations. All atomic positions were fully relaxed using the conjugate gradient algorithm method until the residual forces were below 0.02 eV $Å^{-1}$. To evaluate the photocatalytic carbon dioxide reduction performance of all models, we calculated the Gibbs free energy according to the formula.

$$\Delta G = \Delta E + \Delta ZPE - T\Delta S$$

where ΔE, ΔZPE, ΔS are the change of total energy, zero-point energy and entropy at 298.15 K, respectively.

S2 Supplementary Figures and Tables

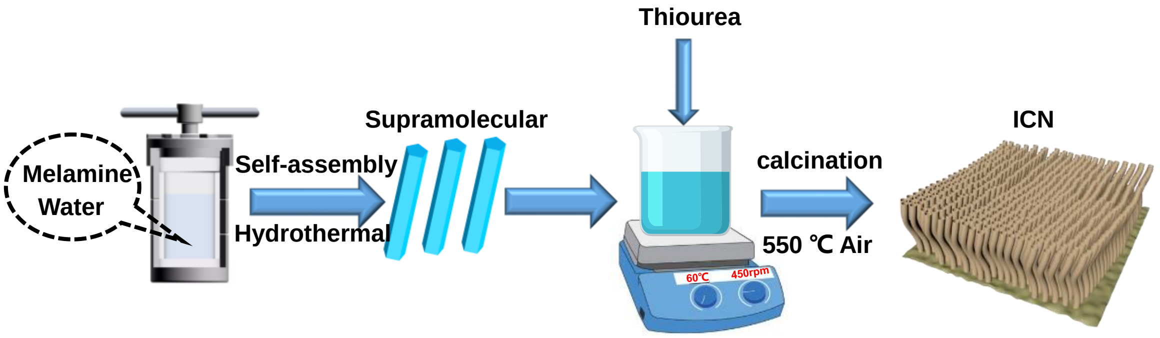

Fig. S1 Schematic illustration for the synthesis of g-C_3N_4 isotype heterojunction (ICN)

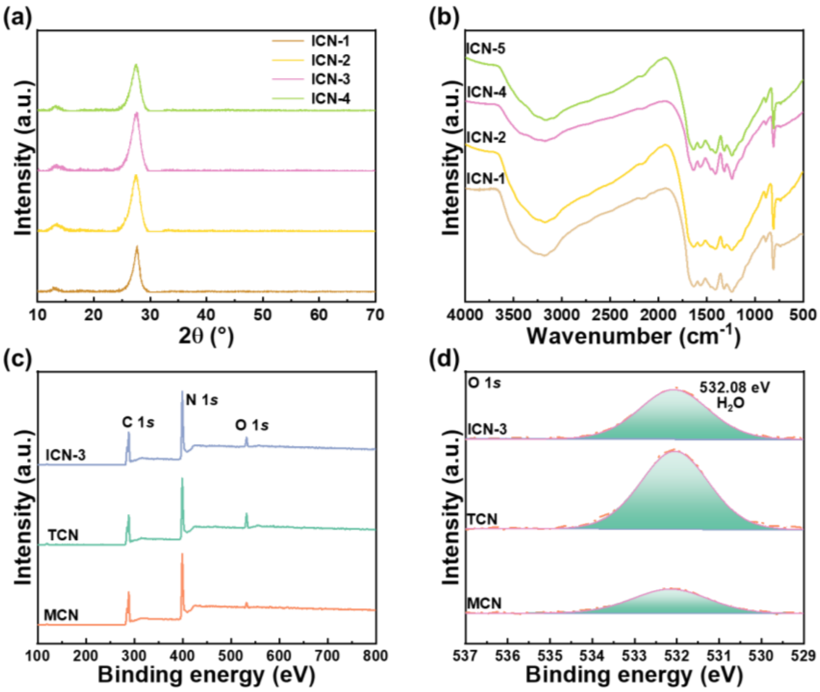

Fig. S2 (**a**) XRD patterns and (**b**) FTIR spectra of ICN-1, ICN-2, ICN-4 and ICN-5. (**c-d**) XPS survey spectra and O 1*s* spectrum of ICN-3, TCN and MCN

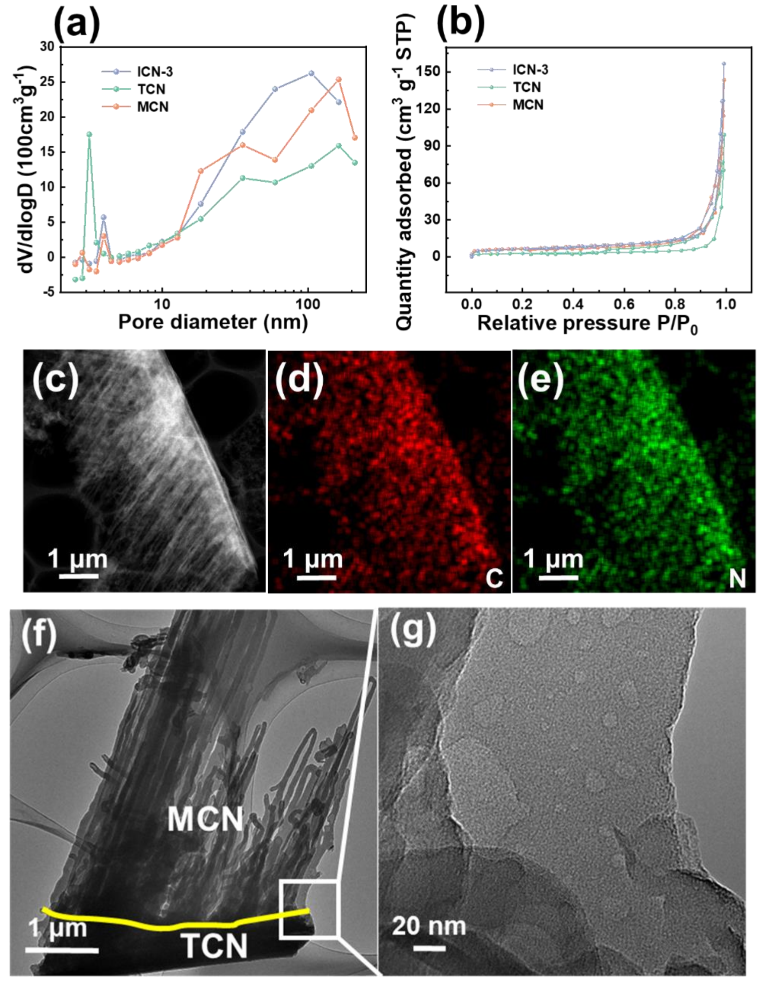

Fig. S3 (**a**) Nitrogen adsorption-desorption isotherms and the (**b**) corresponding pore size distribution curves of ICN-3, TCN and MCN. (**c-f**) HAADF–STEM, the energy dispersive X-ray spectroscopy (EDX) elemental mapping, TEM and HRTEM images of ICN-3

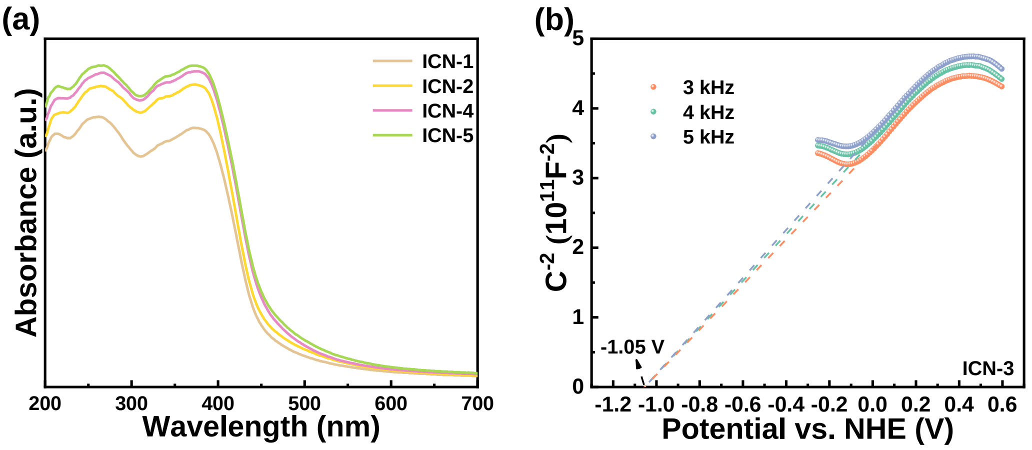

Fig. S4 (**a**) UV-DRS spectra of ICN-1, ICN-2, ICN-4 and ICN-5. (**b**) The Mott-Schottky plots of ICN-3

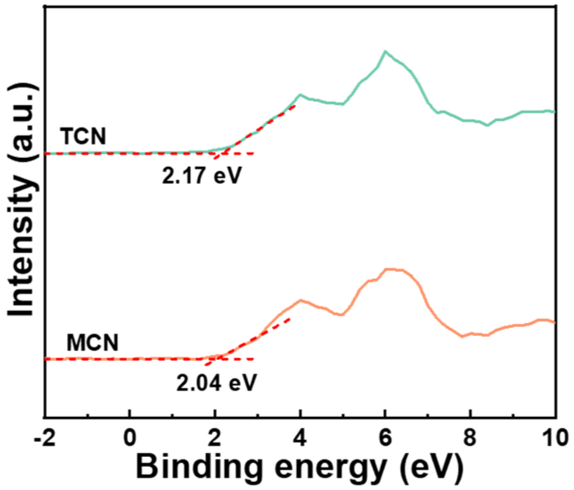

Fig. S5 XPS-VB spectra of TCN and MCN

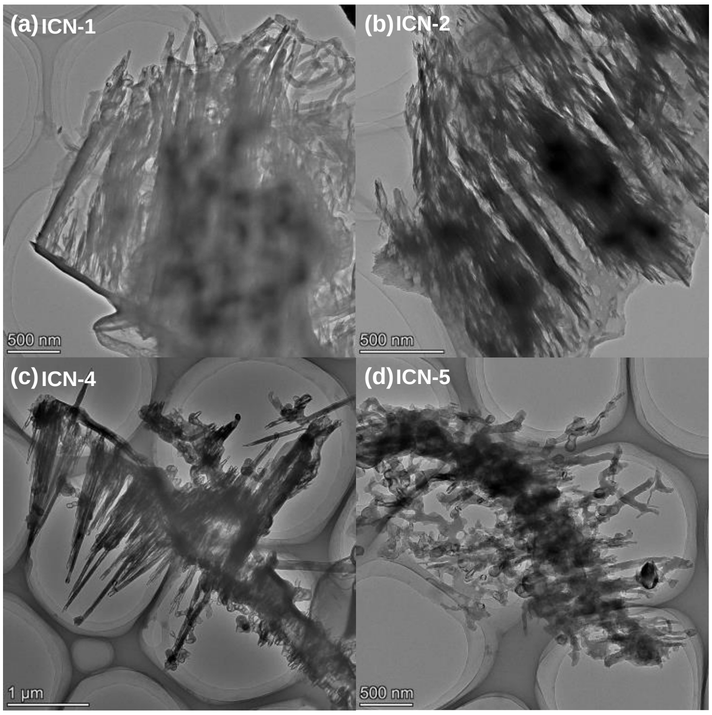

Fig. S6 TEM images of (**a**) ICN-1, (**b**) ICN-2, (**c**) ICN-4 and (**d**) ICN-5

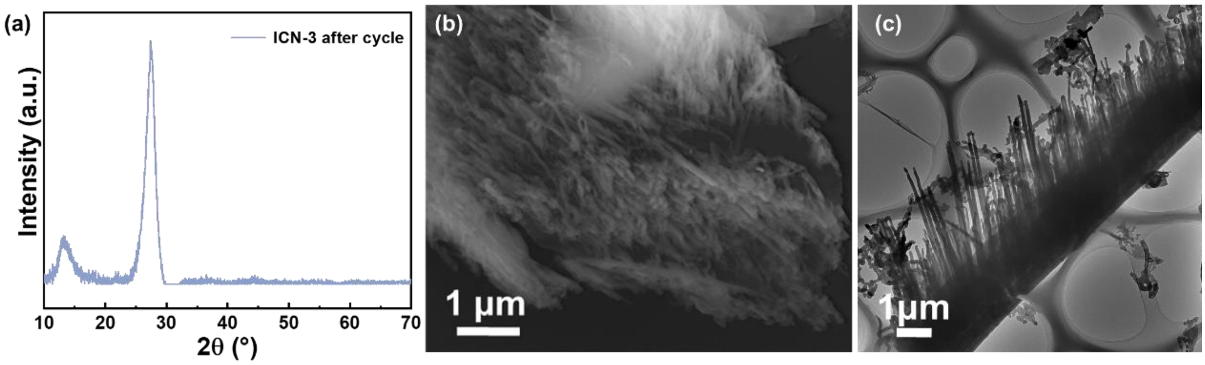

Fig. S7 (**a**) XRD pattern (**b**) SEM and (**c**) TEM images of ICN-3 after 960 min cycle test

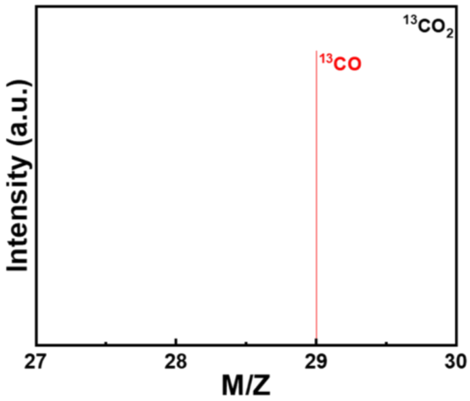

Fig. S8 GC-MS analysis of the products using ^{13}C as the carbon source

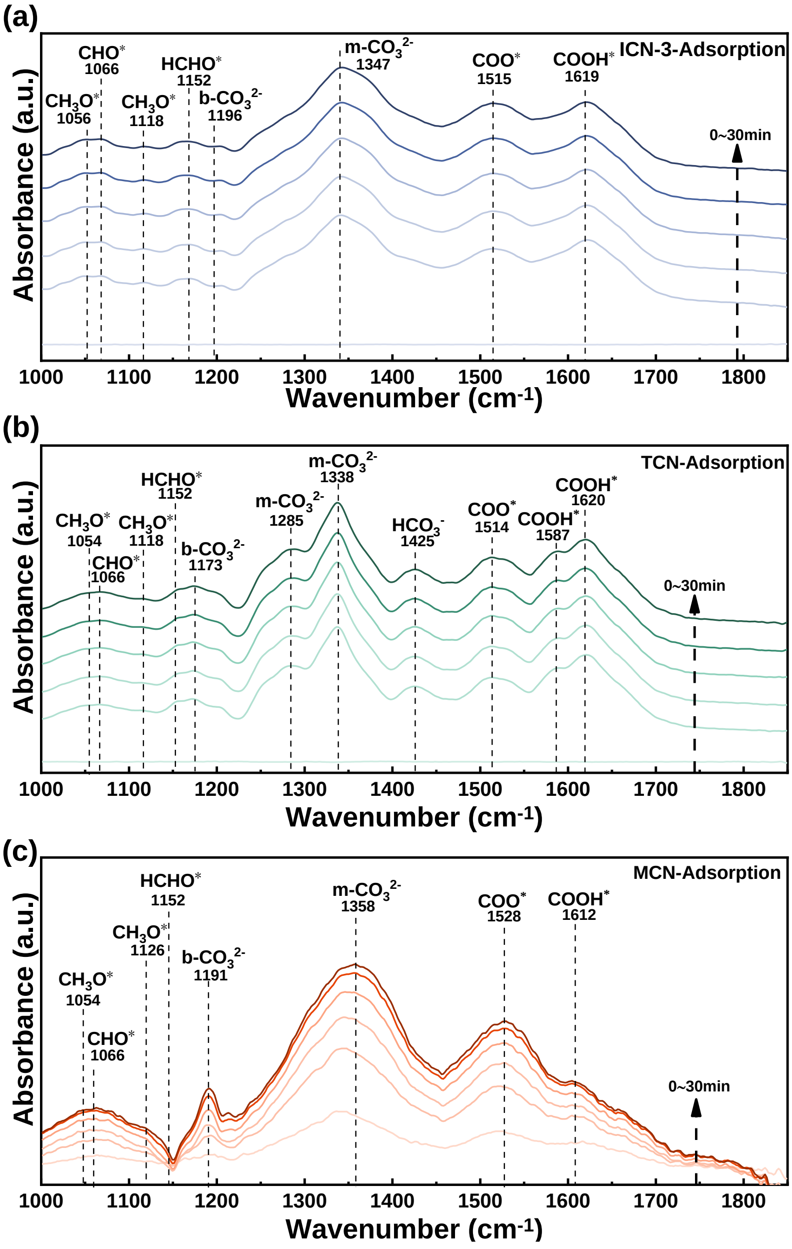

Fig. S9 *In situ* DRIFTS of (**a**) ICN-3, (**b**) TCN and (**c**) MCN during adsorption

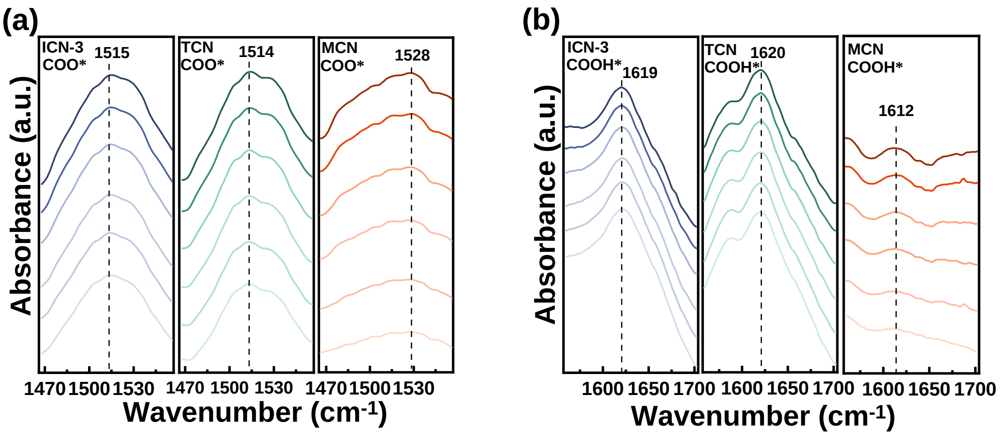

Fig. S10 The local *in situ* DRIFTS analysis of (**a**) *COO and (**b**) *COOH over ICN-3, TCN and MCN during reaction

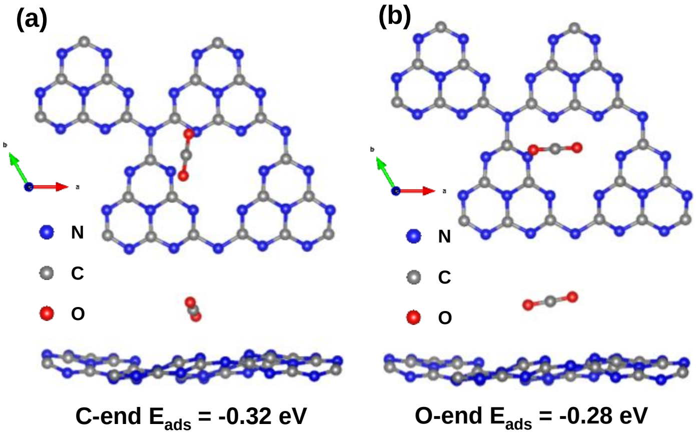

Fig. S11 The CO_2 adsorption on the (**a**) C-end and (**b**) O-end

Table S1 BET surface area and average pore diameter of ICN-3, TCN and MCN

Sample	BET (m^2 g^{-1})	Average diameter of pores (nm)
ICN-3	22.23	46.13
TCN	9.38	29.30
MCN	23.00	52.46

Table S2 Comparison of photocatalytic CO_2 reduction

Catalysts	Catalytic condition	Sacrificial agents	The source of CO_2	CO yield (μmol g^{-1} h^{-1})	Year	Refs.
g-C_3N_4 isotype heterojunction	300 W Xe lamp (AM 1.5)	None	Highly pure CO_2 (99.999%)	12.09	2021	This work
CeO_2/g-C_3N_4	300 W Xe lamp	None	High purity CO_2 gas	4.18	2021	[S8]
Bi_3O_4Cl/g-C_3N_4	300 W Xe lamp (λ > 400 nm)	None	1 ml of CO_2 gas	6.60	2021	[S9]
$Cs_2AgBiBr_6$@g-C_3N_4	simulate solar light	None	1.2 atm pure CO_2	2.00	2021	[S10]
g-C_3N_4 nanotubes/graphdiyne	300 W Xe lamp (λ > 400 nm)	None	High purity CO_2 gas	7.33	2021	[S11]
Potassium-Doped g-C_3N_4	CEAULIGHT CEL-HXF300 with 420 nm cutoff filter	None	High purity CO_2 gas	8.70	2020	[S12]
N defect g-C_3N_4	ultraviolet (UV) lamp (254 nm, 40 μW/cm^2, 4 W)	None	High purity CO_2 gas	8.22	2020	[S13]
Ti_3C_2 MXene/g-C_3N_4	visible light (λ≥420 nm)	None	1.26 g of $NaHCO_3$ react with 4 mL of H_2SO_4 solution (2 M)	5.19	2020	[S14]
Co-MOF/g-C_3N_4	300 W Xe lamp (λ≥420 nm)	None	High-purity CO_2	3.41	2020	[S15]
Bi_4NbO_8Cl/g-C_3N_4	300W Xe lamp, λ > 420 nm	None	1.7 g of $NaHCO_3$ react with 15 mL of	2.26	2020	[S16]

			H_2SO_4 solution (1.1 M)			
Single Ni atoms/g-C_3N_4	visible-light	None	High-purity CO_2	8.60	2020	[S17]
Single Cu atoms/crystalline g-C3N4	xenon lamp	None	High-purity CO_2	3.09	2020	[S18]
g-C_3N_4/$FeWO_4$	300 W xenon lamp with a solar simulator	20 mL of 0.5 M Na_2SO_3	high purity CO2	6.12	2019	[S19]
g-C_3N_4@CeO_2	visible light	None	Ultrahigh-purity CO_2 (99.99%)	4.02	2019	[S20]
CoZnAl-LDH/RGO/g-C_3N_4	300W Xenon	None	High purity of CO_2	10.11	2019	[S21]
g-C_3N_4/NiAl-LDH	300 W Xenon lamp, λ > 420 nm	None	High purity of CO_2	8.20	2018	[S22]
NiO/g-C_3N_4	300W Xenon lamp, visible-light	None	100 kPa CO_2 (99.999%)	4.17	2018	[S23]
Phosphorus-doped g-C_3N_4	300W Xenon lamp	triethanolamine	1.3 g $NaHCO_3$ react with 5 mL of H_2SO_4 solution (4 M)	2.37	2018	[S24]
MnO_2/g-C_3N_4	A 300 W Xe lamp	None	High purity CO_2 (99.99%)	3.40	2017	[S25]

Eq. S1

Charge transfer rate: $k_{ET}=\frac{1}{\tau_{ave(ICN-3)}}-\frac{1}{\tau_{ave(TCN)}}$ (S1)

Eq. S2

Charge transfer efficiency: $\eta_{ET}=1-\frac{\tau_{ave(ICN-3)}}{\tau_{ave(TCN)}}$ (S2)

Supplementary References

[S1] G. Kresse, J. Furthmuller, Efficient iterative schemes for ab initio total-energy calculations using a plane-wave basis set. Phys. Rev. B **54**(16), 11169-11186 (1996). https://doi.org/10.1103/PhysRevB.54.11169

[S2] G. Kresse, J. Furthmuller, Efficiency of ab-initio total energy calculations for metals and semiconductors using a plane-wave basis set. Comp. Mater. Sci. **6**(1), 15-50 (1996). https://doi.org/10.1016/0927-0256(96)00008-0

[S3] C. Filippi, D.J. Singh, C.J. Umrigar, All-electron local-density and generalized-gradient calculations of the structural-properties of semiconductors. Phys. Rev. B **50**(20), 14947-14951 (1994). https://doi.org/10.1103/PhysRevB.50.14947

[S4] J.P. Perdew, K. Burke, M. Ernzerhof, Generalized gradient approximation made simple. Phys. Rev. Lett. **77**(18), 3865-3868 (1996). https://doi.org/10.1103/PhysRevLett.77.3865

[S5] S. Grimme, J. Antony, S. Ehrlich, H. Krieg, A consistent and accurate ab initio parametrization of density functional dispersion correction (DFT-D) for the 94 elements H-Pu. J. Chem. Phys. **132**(15), 154104 -154124 (2010). https://doi.org/10.1063/1.3382344

[S6] B. Amin, N. Singh, U. Schwingenschlögl, Heterostructures of transition metal dichalcogenides. Phys. Rev. B **92**(7), 075439 (2015). https://doi.org/10.1103/PhysRevB.92.075439

[S7] M. Lucking, Y.Y. Sun, D. West, S.B. Zhang, A nucleus-coupled electron transfer mechanism for TiO_2-catalyzed water splitting. Phys. Chem. Chem. Phys. **17**(26), 16779-16783 (2015). https://doi.org/10.1039/C5CP01202C

[S8] W.Q. Li, L. Jin, F. Gao, H.Q. Wan, Y. Pu et al., Advantageous roles of phosphate decorated octahedral CeO_2 {111}/g-C_3N_4 in boosting photocatalytic CO_2 reduction: charge transfer bridge and Lewis basic site. Appl. Catal. B **294**, 120257 (2021). https://doi.org/10.1016/j.apcatb.2021.120257

[S9] Y.X. Xu, X.L. Jin, T. Ge, H.Q. Xie, R.X. Sun et al., Realizing efficient CO_2 photoreduction in Bi_3O_4Cl: constructing van der Waals heterostructure with g-C_3N_4. Chem. Eng. J. **409**, 128178 (2021). https://doi.org/10.1016/j.cej.2020.128178

[S10] Y.Y. Wang, H.L. Huang, Z.Z. Zhang, C. Wang, Y.Y. Yang et al., Lead-free perovskite $Cs2AgBiBr_6$@g-C_3N_4 Z-scheme system for improving CH_4 production in photocatalytic CO_2 reduction. Appl. Catal. B **282**, 119570 (2021). https://doi.org/10.1016/j.apcatb.2020.119570

[S11] C. Sun, Y.Y. Liu, Z.Y. Wang, P. Wang, Z.K. Zheng et al., Self-assembled g-C_3N_4 nanotubes/graphdiyne composite with enhanced photocatalytic CO_2 reduction. J. Alloys Compd. **868**, 159045 (2021). https://doi.org/10.1016/j.jallcom.2021.159045

[S12] S.H. Wang, J.W. Zhan, K. Chen, A. Ali, L.H. Zeng et al., Potassium-doped g-C_3N_4 achieving efficient visible-light-driven CO_2 reduction. ACS Sustain. Chem. Eng. **8**(22), 8214-8222 (2020). https://doi.org/10.1021/acssuschemeng.0c01151

[S13] X.H. Song, X.Y. Zhang, X. Li, H.N. Che, P.W. Huo et al., Enhanced light utilization efficiency and fast charge transfer for excellent CO_2 photoreduction activity by constructing defect structures in carbon nitride. J. Colloid Interface Sci. **578**, 574-583 (2020). https://doi.org/10.1016/j.jcis.2020.06.035

[S14] C. Yang, Q.Y. Tan, Q. Li, J. Zhou, J.J. Fan et al., 2D/2D Ti_3C_2 MXene/g-C_3N_4 nanosheets heterojunction for high efficient CO_2 reduction photocatalyst: dual effects of urea. Appl. Catal. B **268**, 118738 (2020). https://doi.org/10.1016/j.apcatb.2020.118738

[S15] Q.Y. Chen, S.J. Li, H.Y. Xu, G.F. Wang, Y. Qu et al., Co-MOF as an electron donor for promoting visible-light photoactivities of g-C_3N_4 nanosheets for CO_2 reduction. Chinese J. Catal. **41**(3), 514-523 (2020). https://doi.org/10.1016/S1872-2067(19)63497-2

[S16] Y. Xu, Y. You, H.W. Huang, Y.X. Guo, Y.H. Zhang, Bi_4NbO_8Cl {001} nanosheets coupled with g-C_3N_4 as 2D/2D heterojunction for photocatalytic degradation and CO_2 reduction. J. Hazard. Mater. **381**, 121159 (2020). https://doi.org/10.1016/j.jhazmat.2019.121159

[S17] L. Cheng, H. Yin, C. Cai, J.J. Fan, Q.J. Xiang, Single Ni atoms anchored on porous few-layer g-C_3N_4 for photocatalytic CO_2 reduction: the role of edge confinement. Small **16**(28), 2002411 (2020). https://doi.org/10.1002/smll.202002411

[S18] Y. Li, B.H. Li, D.N. Zhang, L. Cheng, Q.J. Xiang, Crystalline carbon nitride supported copper single atoms for photocatalytic CO_2 reduction with nearly 100% CO selectivity. ACS Nano **14**(8), 10552-10561 (2020).

https://doi.org/10.1021/acsnano.0c04544

[S19] R. Bhosale, S. Jain, C.P. Vinod, S. Kumar, S. Ogale, Direct Z-scheme g-C_3N_4/$FeWO_4$ nanocomposite for enhanced and selective photocatalytic CO_2 reduction under visible light. ACS Appl. Mater. Interfaces **11**(6), 6174-6183 (2019). https://doi.org/10.1021/acsami.8b22434

[S20] M.F. Liang, T. Borjigin, Y.H. Zhang, B.H. Liu, H. Liu et al., Controlled assemble of hollow heterostructured g-C_3N_4@CeO_2 with rich oxygen vacancies for enhanced photocatalytic CO_2 reduction. Appl. Catal. B **243**, 566-575 (2019). https://doi.org/10.1016/j.apcatb.2018.11.010

[S21] Y. Yang, J.J. Wu, T.T. Xiao, Z. Tang, J.Y. Shen et al., Urchin-like hierarchical CoZnAl-LDH/RGO/g-C_3N_4 hybrid as a Z-scheme photocatalyst for efficient and selective CO_2 reduction. Appl. Catal. B **255**, 117771 (2019). https://doi.org/10.1016/j.apcatb.2019.117771

[S22] S. Tonda, S. Kumar, M. Bhardwaj, P. Yadav, S. Ogale, g-C_3N_4/NiAl-LDH 2D/2D hybrid heterojunction for high-performance photocatalytic reduction of CO_2 into renewable fuels. ACS Appl. Mater. Interfaces **10**(3), 2667-2678 (2018). https://doi.org/10.1021/acsami.7b18835

[S23] J.Y. Tang, R.T. Guo, W.G. Zhou, C.Y. Huang, W.G. Pan, Ball-flower like NiO/g-C_3N_4 heterojunction for efficient visible light photocatalytic CO_2 reduction. Appl. Catal. B **237**, 802-810 (2018). https://doi.org/10.1016/j.apcatb.2018.06.042

[S24] B. Liu, L.Q. Ye, R. Wang, J.F. Yang, Y.X. Zhang et al., Phosphorus-doped graphitic carbon nitride nanotubes with amino-rich surface for efficient CO_2 capture, enhanced photocatalytic activity, and product selectivity. ACS Appl. Mater. Interfaces **10**(4), 4001-4009 (2018). https://doi.org/10.1021/acsami.7b17503

[S25] M. Wang, M. Shen, L.X. Zhang, J.X. Tian, X.X. Jin et al., 2D-2D MnO_2/g-C_3N_4 heterojunction photocatalyst: in-situ synthesis and enhanced CO_2 reduction activity. Carbon **120**, 23-31 (2017). https://doi.org/10.1016/j.carbon.2017.05.024
